# Plasma Vitamin C and Cancer Mortality in Kidney Transplant Recipients

**DOI:** 10.3390/jcm8122064

**Published:** 2019-11-23

**Authors:** Tomás A. Gacitúa, Camilo G. Sotomayor, Dion Groothof, Michele F. Eisenga, Robert A. Pol, Martin H. de Borst, Rijk O.B. Gans, Stefan P. Berger, Ramón Rodrigo, Gerjan J. Navis, Stephan J.L. Bakker

**Affiliations:** 1 Department of Internal Medicine, University Medical Center Groningen, University of Groningen, 9713 GZ Groningen, The Netherlands; t.a.gacitua.guzman@umcg.nl (T.A.G.); d.groothof@umcg.nl (D.G.); m.f.eisenga@umcg.nl (M.F.E.); m.h.de.borst@umcg.nl (M.H.d.B.); s.p.berger@umcg.nl (S.P.B.); g.j.navis@umcg.nl (G.J.N.);; 2 Department of Surgery, University Medical Center Groningen, University of Groningen, 9713 GZ Groningen, The Netherlands; r.pol@umcg.nl; 3 Molecular and Clinical Pharmacology Program, Institute of Biomedical Sciences, Faculty of Medicine, University of Chile, CP 8380453 Santiago, Chile; rrodrigo@med.uchile.cl

**Keywords:** Kidney transplant, vitamin C, cancer mortality, oxidative stress.

## Abstract

There is a changing trend in mortality causes in kidney transplant recipients (KTR), with a decline in deaths due to cardiovascular causes along with a relative increase in cancer mortality rates. Vitamin C, a well-known antioxidant with anti-inflammatory and immune system enhancement properties, could offer protection against cancer. We aimed to investigate the association of plasma vitamin C with long-term cancer mortality in a cohort of stable outpatient KTR without history of malignancies other than cured skin cancer. Primary and secondary endpoints were cancer and cardiovascular mortality, respectively. We included 598 KTR (mean age 51 ± 12 years old, 55% male). Mean (SD) plasma vitamin C was 44 ± 20 μmol/L. At a median follow-up of 7.0 (IQR, 6.2–7.5) years, 131 patients died, of which 24% deaths were due to cancer. In Cox proportional hazards regression analyses, vitamin C was inversely associated with cancer mortality (HR 0.50; 95%CI 0.34–0.74; *p* < 0.001), independent of potential confounders, including age, smoking status and immunosuppressive therapy. In secondary analyses, vitamin C was not associated with cardiovascular mortality (HR 1.16; 95%CI 0.83–1.62; *p* = 0.40). In conclusion, plasma vitamin C is inversely associated with cancer mortality risk in KTR. These findings underscore that relatively low circulating plasma vitamin C may be a meaningful as yet overlooked modifiable risk factor of cancer mortality in KTR.

## 1. Introduction

Although kidney transplantation improves the prognosis of patients with end-stage renal disease (ESRD), kidney transplant recipients (KTR) remain at higher mortality risk compared to healthy individuals [1]. Since the beginning of kidney transplantation, the main cause of death has been cardiovascular [2,3,4]. In recent years, however, there has been a changing trend in mortality causes in KTR, with a decline in death due to cardiovascular causes along with a relative increase in cancer mortality [2,5,6,7]. Among non-cardiovascular deaths, malignancies lead the individual causes of death [8,9]. Noteworthy is that overall risk of death associated with cancer in KTR is ten-fold higher than in the general population [9]. Given this relative increase in cancer mortality in KTR, further studies to explore potential risk factors and underlying mechanisms are needed.

Post-transplantation immunosuppression as well as chronic uremic state have been recently proposed as risk factors, with oxidative stress as a potential underlying mechanism [2,10,11]. Vitamin C is a well-known radical scavenger and reducing agent [12], and due to its antioxidant, anti-inflammatory and immune system enhancement properties, it could offer protection against cancer incidence in KTR [13]. There is evidence supporting that low plasma vitamin C may lead to an increased risk of dying from cancer in the general male population [13], and is also inversely associated with gastric cancer risk in the general population [14].

Increased oxidative stress occurs when there is an imbalance between antioxidant and pro-oxidant species, leading to oxidative damage. Malondialdehyde (MDA), a decomposition product of peroxidized polyunsaturated fatty acids, is a widely used and sensitive biomarker of oxidative damage [15]. Gamma-glutamyl transpeptidase (GGT) is also currently used as an indicator of whole body oxidative stress [16,17]. Uric acid in plasma acts as antioxidant in presence of vitamin C [18]. Higher levels of free thiol groups have been proposed to be protective against oxidative damage, similarly to vitamin C [19]. Under the hypothesis that anti-carcinogenic properties of vitamin C are mainly driven by its antioxidant properties, the potential protective effect of vitamin C against cancer mortality would be expected to vary upon changes in oxidative stress biomarkers.

This evidence suggests that vitamin C could be a simple and widely available modifiable risk factor for cancer mortality in KTR. Nevertheless, studies focusing on the prospective association of vitamin C and long-term cancer mortality in this clinical setting are lacking. In this study, in primary analyses we aimed to investigate the association of circulating plasma vitamin C concentrations with long-term cancer mortality in a large cohort of KTR. As oxidative stress is considered a potential underlying mechanism, we aimed to assess whether the potential association of plasma vitamin C with cancer mortality would vary upon changes in oxidative stress biomarkers, i.e., uric acid, free thiol groups, MDA and GGT. In secondary analyses, we aimed to investigate the association of circulating plasma vitamin C concentrations with cardiovascular mortality.

## 2. Materials and Methods

### 2.1. Study Design and Patients

We performed a post hoc analysis in the TransplantLines Insulin Resistance and Inflammation Biobank and Cohort Study, number NCT03272854. Outpatient KTR (≥18 years old) with a functioning graft for at least 1 year were invited to participate between August 2001 and July 2003. Patients with overt congestive heart failure and patients diagnosed with cancer other than cured skin cancer (squamous cell or basal cell carcinoma successfully treated by a dermatologist) were not considered eligible for the study. The outpatient follow-up constitutes a continuous surveillance system in which patients visit the outpatient clinic with declining frequency, in accordance with the American Transplantation Society guidelines [20]. A total of 847 KTR were invited to be enrolled, of which 606 (72%) patients provided written informed consent to participate. Data were extensively collected at baseline. Patients with missing plasma vitamin C concentration (*n* = 8) were excluded for the statistical analysis, resulting in 598 KTR, of whom data are presented in the current study (Figure S1). The present study was approved by the Institutional Review Board (METc 2001/039), and was conducted in accordance with declarations of Helsinki and Istanbul.

### 2.2. Kidney Transplant Recipients Characteristics

Relevant characteristics including recipient age, gender, and transplant date were extracted from the Groningen Renal Transplant Database. This database contains detailed information on all kidney transplantations that have been performed at the University Medical Center Groningen since 1968. Details of the standard immunosuppressive treatment were described previously [21]. Smoking status was obtained using a self-report questionnaire at inclusion. Details about collection of dietary history have been described before [22]. In brief, a semi-quantitative food-frequency questionnaire was used to assess fruit and vegetable intake. Fruit intake was assessed by asking participants ‘How many servings of fruit do you eat per day on average?’ Vegetable intake was assessed by asking participants ‘How many tablespoons of vegetable do you eat per day on average?’ Respondents were asked to choose among five possible frequency categories: 0, 1, 2, 3, ≥4 per day. Collection of data on use of vitamin C or multivitamin supplements containing vitamin C was systematically performed, by means of self-report, at baseline.

### 2.3. Laboratory Measurements

All measurements were performed during a morning visit to the outpatient clinic. Diabetes mellitus was defined according to the guidelines of the American Diabetes Association [23]. Proteinuria was defined as urinary protein excretion ≥0.5 g/24 h. Kidney function was assessed by estimated Glomerular Filtration Rate (eGFR) applying the Chronic Kidney Disease Epidemiology Collaboration equation [24].

Blood was drawn after a fasting period of 8–12 h, which included no medication intake. According to a strict protocol, patients were instructed to collect a 24-hour urine sample the day before their visit to the outpatient clinic. Total cholesterol, low-density lipoprotein cholesterol (LDL), plasma triglycerides, plasma glucose levels, plasma insulin concentration, and glycated hemoglobin (HbA_1C_) were determined as described previously [25]. Plasma high sensitivity C-reactive protein (hs-CRP) was measured by enzyme-linked immunosorbent assay, as described previously [26]. MDA was measured fluorescently after binding to thiobarbituric acid as described before [27]. Ellman’s reagent was used for the determination of free thiol groups in cell culture and a cell-free solution of L-cysteine as described previously [28]. Plasma creatinine concentration was determined using a modified version of the Jaffé method (MEGA AU510; Merck Diagnostica). Total urinary protein concentration was analyzed using the Biuret reaction (MEGA AU510; Merck Diagnostica).

### 2.4. Plasma Vitamin C Measurement

After phlebotomy, blood was directly transferred to the laboratory on ice, deproteinized and stored in the dark at −20°C until analysis. For quantitative measurement ascorbic acid is enzymatically transformed to dehydroascorbic acid, which in turn is derivatized to 3-(1,2-dihydroxyethyl) furo-[3,4-b] quinoxaline-1-one. Then, reversed phase liquid chromatography with fluorescence detection is applied (excitation 355 nm, emission 425 nm).

### 2.5. Cause-Specific Mortality and Graft Failure

The primary endpoint for analyses was mortality from cancer, defined according to a previously specified list of International Classification of Diseases, Ninth Revision (ICD-9) codes 140–239 [29]. Secondary endpoint was mortality from cardiovascular causes, defined as death due to cerebrovascular disease, ischemic heart disease, heart failure, or sudden cardiac death according to ICD-9 codes 410–447. Information on the cause of death was derived from the patients’ medical records and was assessed by an adjudication committee. Information about death-related type of cancer was ascertained by contacting the general practitioners who were in charge of deceased cancer patients. Graft failure was defined as return to dialysis or need for a re-transplantation. The continuous surveillance system of the outpatient program ensures up-to-date information on patient status and cause of death. There was no loss to follow-up.

### 2.6. Statistical Analyses

Data analysis was performed using SPSS version 23.0 software (SPSS Inc., Chicago, IL, USA), STATA 14.1 (STATA Corp., College Station, TX, USA), and R version 3.2.3 (R Foundation for Statistical Computing, Vienna, Austria). In all analyses, a two-sided *p* < 0.05 was considered significant. Continuous variables were summarized using mean (standard deviation; SD) for normally distributed data, whereas skewed distributed variables are given as median (interquartile range; IQR). Categorical variables were summarized as numbers (percentage). Multiple imputation was performed to account for missingness of data among variables other than data on plasma vitamin C [30]. The percentages of missing data were 0.2, 0.2, 0.2, 0.2, 0.3, 0.3, 0.3, 0.3, 0.5, 0.7, and 0.7% for waist circumference, HbA_1C_, albumin, alkaline phosphatase, proteinuria, leukocyte concentration, MDA, cumulative dose of prednisolone, uric acid, GGT, and prior history of cardiovascular disease, respectively. The percentages of missing data were maximally 11, 21, and 33% for free thiol groups, free fatty acids, and fruit and vegetable intake, respectively.

Age- and sex-adjusted linear regression analyses were performed to evaluate the association of plasma vitamin C concentrations with baseline characteristics. Residuals were checked for normality and variables were natural log-transformed when appropriate. In order to study in an integrated manner which patient- and transplant-related variables of interest were independently associated with and were determinants of plasma vitamin C concentrations, we performed forward selection of baseline characteristics by including all the variables that were associated with plasma vitamin C with a *p* < 0.1 in the preceding age- and sex-adjusted linear regression analyses. Selected variables were then used to perform stepwise backwards multivariable linear regression analyses (*P_out_ >* 0.05). Standardized beta coefficients represent the difference (in standard deviations) in plasma vitamin C per 1 standard deviation increment in continuous baseline characteristics, or for categorical characteristics the difference (in standard deviations) in plasma vitamin C compared to the implied reference group.

To analyze whether plasma vitamin C was prospectively and independently associated with cancer mortality, we performed multivariable-adjusted Cox proportional hazards regression analyses. For these analyses plasma vitamin C concentrations were used as log-transformed values with a log2 base, in order to obtain the best fitting model. We tested proportionality assumptions of Cox proportional hazards regression analyses, and they were satisfied, indicating that the association of baseline vitamin C with outcome is constant over follow-up time of the current study. The selection of covariates was made a priori, considering their potential confounding effect based on previously described risk factors for all-cause mortality in KTR and generally accepted risk factors for cancer mortality in the general population and in KTR [9,10,13,31]. We adjusted for age, sex, and smoking status (Model 1); eGFR, dialysis vintage, time since transplantation and proteinuria (Model 2); and, fruit and vegetable intake (Model 3). To avoid overfitting and inclusion of too many variables for the number of events, further models were performed with additive adjustments to Model 3 [32]. We performed additional adjustments for diabetes mellitus, hs-CRP and prior history of cardiovascular disease (Model 4); immunosuppressive therapy (use of calcineurin inhibitors (CNI), use of antimetabolites, use of mammalian target of rapamycin (m-TOR) inhibitors, and cumulative dose of prednisolone, calculated as the sum of maintenance dose of prednisolone since kidney transplantation until inclusion in the study and the dose of prednisolone or methylprednisolone required for treatment of acute rejection (a conversion factor of 1.25 was used to convert methylprednisolone to prednisolone dose). For acute rejection, different amounts of prednisolone or methylprednisolone were administered, which was taken into account in the calculations. Rejection episodes after inclusion were not included [33]; Model 5); and transplantation era (Model 6). Transplantation eras, with corresponding immunosuppressing medications, have been previously well described [34]. In secondary analyses, the aforementioned Cox proportional hazards regression analyses were performed for cardiovascular mortality. The analyses for both cancer death and cardiovascular death were performed by fitting multivariable-adjusted proportional cause-specific hazard models. In each of these models, the competing events were treated as censored observations, causing the regression parameters to directly quantify the hazard ratio among those individuals who are actually at risk of developing the event of interest, i.e., cancer mortality or cardiovascular mortality [35]. Hazard ratios (HR) are reported with 95% confidence interval (CI). The HR of each model is given per doubling of vitamin C concentration.

To adhere to existing recommendations for good reporting on survival analyses [36,37], we tested for potential interaction of all potential confounders and the oxidative stress biomarkers with vitamin C, namely, uric acid, free thiol groups (corrected by total serum protein) [19], MDA, and GGT by fitting models containing both main effects and their cross product terms. For these analyses, *P*_interaction_ < 0.05 was considered to indicate significant interaction. We also performed subgroup analyses according to the aforementioned oxidative stress biomarkers, with adjustment for age, sex, smoking status, eGFR, dialysis vintage, time since transplantation, proteinuria, and fruit and vegetable intake. Cut-off points of originally continuous variables used in the stratified analyses were determined so they would allow for an as much as possible similar number of events in each subgroup, and thus allow for similar statistical power for the assessment of the primary association under study (plasma vitamin C and cancer mortality) in each subgroup after stratification of the overall population. Whenever and as much as possible, these criteria were matched with clinical cut-off points.

In sensitivity analyses, we performed graft failure-censored Cox proportional hazards regression analyses of the association of plasma vitamin C with cancer mortality and cardiovascular mortality. In addition, we performed Cox proportional hazards regression analyses of the association of plasma vitamin C with cancer mortality with adjustment for HbA1c instead of diabetes mellitus.

## 3. Results

### 3.1. Baseline Characteristics

A total of 598 patients (51 ± 12 years old, 55% male) were included at a median of 5.9 (IQR, 2.6–11.4) years after kidney transplantation. None of the patients used vitamin C supplements or multivitamin supplements containing vitamin C. Mean plasma vitamin C concentration was 44 ± 20 μmol/L, mean eGFR was 47 ± 16 mL/min/1.73 m^2^. Patient-related variables of interest, including transplant-related characteristics and immunosuppressive therapy are summarized in Table 1. The results of the age- and sex-adjusted linear regression analyses are shown in Table 2. In stepwise backward multivariable linear regression analysis, fruit intake (std. β = 0.22; *p* < 0.01), dialysis vintage (std. β = −0.09; *p* < 0.05), proteinuria ≥0.5 g/24 h (std. β = −0.11; *p* < 0.05), HbA_1C_ (std. β = −0.14; *p* < 0.01), diastolic blood pressure (std. β = −0.16; *p* < 0.01), alkaline phosphatase (std. β = −0.15; *p* < 0.01), hs-CRP (std. β = −0.17; *p* < 0.01) and male sex (std. β = −0.18; *p* < 0.01) were identified as independent determinants of plasma vitamin C (Table 2). The overall *R*^2^ of the final model was 0.21.

### 3.2. Primary Prospective Analyses

**Figure 1:**
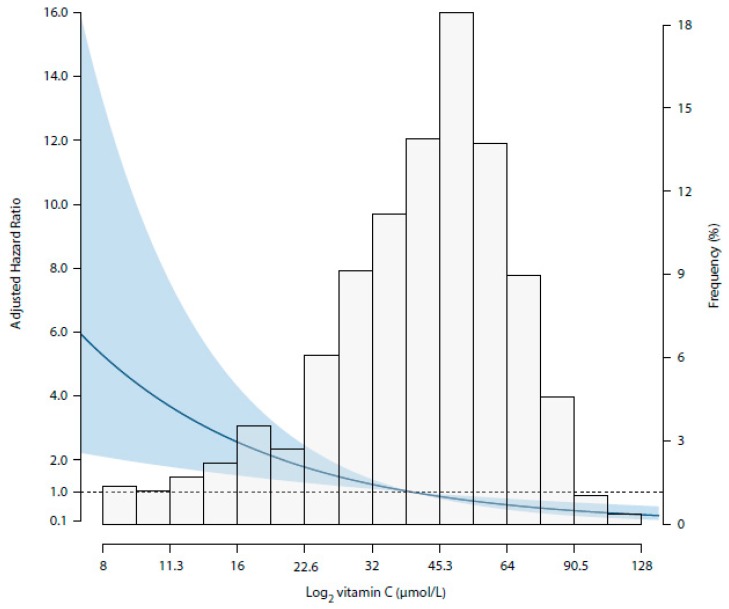
Association of plasma vitamin C with cancer mortality risk in 598 KTR. Data were fitted by a Cox proportional hazards regression model adjusted for age, sex, smoking status, estimated Glomerular Filtration Rate, dialysis vintage, time since transplantation, proteinuria, fruit and vegetable intake, diabetes mellitus, high-sensitivity C-reactive protein, and prior history of cardiovascular disease (Model 4). The gray areas indicate the 95% CIs. The line in the graph represents the hazard ratio.

**Figure 2:**
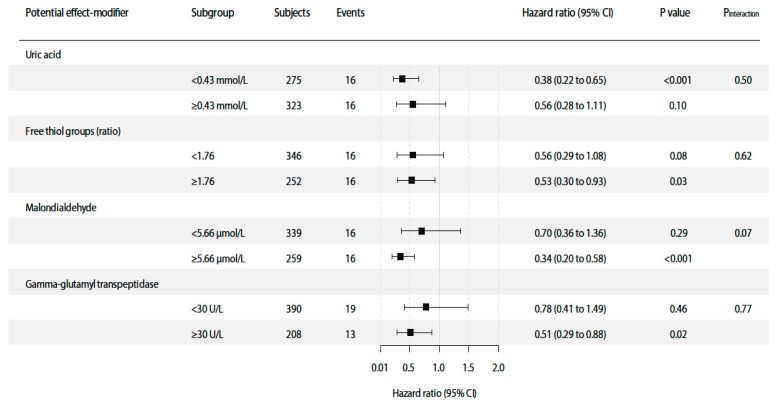
Interaction and subgroup analyses of the association of plasma vitamin C with cancer mortality. *P*_interaction_ was calculated by fitting models which contain both main effects as continuous variables and their cross-product term. Hazard ratios were calculated with adjustment for age, sex, smoking status, estimated Glomerular Filtration Rate, dialysis vintage, time since transplantation, proteinuria, and fruit and vegetable intake, analogous to Model 3 of the overall prospective analyses. Abbreviations: CI, confidence interval; MDA, malondialdehyde; GGT, gamma-glutamyl transpeptidase.

At a median follow-up of 7.0 (IQR, 6.2–7.5) years, 131 (22%) patients died, of which 32 (24%) deaths were due to cancer (summary of types of cancer can be found in Table S1). Median time from kidney transplantation to cancer death was 12.0 (IQR, 6.2–20.0). In multivariable-adjusted Cox proportional hazards regression analyses, plasma vitamin C concentration was inversely associated with cancer mortality risk (HR 0.50; 95%CI 0.34–0.74; *p* < 0.001), independent of potential confounders including age, sex, smoking status, eGFR, dialysis vintage, time since transplantation, proteinuria, fruit and vegetable intake, diabetes mellitus, hs-CRP, prior history of cardiovascular disease, immunosuppressive therapy and transplantation era (Table 3, Models 1–6) (Figure 1). Full report of coefficient estimates for both the variable of interest plasma vitamin C as well as for potential confounders included in every multivariable model (Models 1–6) are shown in Table S2. Neither significant interaction of the association of vitamin C with cancer mortality was found for potential confounders (Table S3) nor for oxidative stress biomarkers. Results of interaction and subgroup analyses of oxidative stress biomarkers are presented in Figure 2.

### 3.3. Secondary Prospective Analyses

In secondary analyses, at a median follow-up of 7.0 (IQR, 6.2–7.5) years, 131 (22%) patients died, of which 67 (49%) deaths were due to cardiovascular causes. Median time from kidney transplantation to cardiovascular death was 11.0 (IQR, 7.6–14.8). There was no significant association of plasma vitamin C with cardiovascular mortality (HR 1.16; 95%CI 0.83–1.62; *p* = 0.40) (Table 4). This finding remained unaltered after adjustment for potential confounders, analogous to Models 1 to 6 of the primary analyses.

### 3.4. Sensitivity Analyses

After performing graft failure-censored Cox proportional hazards regression analyses, our primary findings of the association of plasma vitamin C with both cancer mortality and cardiovascular mortality remained materially unchanged (Tables S4 and S5, respectively). After performing Cox proportional hazards regression analyses of the association of plasma vitamin C with cancer mortality with adjustment for HbA1c instead of diabetes mellitus the association remained materially unchanged (Table S6).

## 4. Discussion

In the current study, we show that cancer is a substantially prevalent individual cause of death after kidney transplantation, and that plasma vitamin C concentrations are inversely and independently associated with long-term cancer mortality risk in stable KTR. Secondary analyses did not reveal significant associations with cardiovascular mortality. To the best of our knowledge, this is the first study that provides prospective data supporting vitamin C as a potential risk factor for cancer mortality in KTR.

Our results are in line with previously reported cancer mortality risk data in KTR. Au et al. reported that 16.7% of deaths in a large cohort of KTR were due to cancer after a median follow-up of 6.3 (IQR, 2.3–12.0) years. Although cancer mortality has been previously described as an increasing and imperative problem in KTR [2,5,6,10], there is a paucity of studies exploring potential risk factors and underlying mechanisms leading to this increased cancer mortality in KTR. Immunosuppression following kidney transplant is the most accepted risk factor, specifically CNI [4,6,38,39]. In fact, there is extensive research focused on finding the best combination of immunosuppressants in order to reduce de novo malignancy incidence without increasing rejection rates, where m-TOR inhibitors could have a role in reducing cancer risk [6,40,41,42]. Noteworthy is that according to our findings, the association of plasma vitamin C concentrations with cancer mortality is independent of immunosuppressive therapies after a kidney transplant.

Low plasma vitamin C has been previously associated with gastric cancer risk in the general population. In this patient setting, mean plasma vitamin C concentration was 39.9 ± 25.2 μmol/L for cases and 41.5 ± 19.4 μmol/L for controls, both comparable to those from our study [14]. Likewise, in the general male population, low plasma vitamin C was linked to an increased risk of mortality with cancer playing a key role. In this study, median plasma vitamin C was 49.4 (IQR, 47.7–51.7) μmol/L [13], also comparable to our study. Furthermore, the anti-cancer properties from vitamin C and other antioxidants have drawn much attention in the oncology research field [43,44,45,46]. According to the results of cross-sectional analyses of our study, daily fruit intake was independently associated with plasma vitamin C levels, congruent with evidence suggesting a diet high in fruits to be associated with decreased cancer risk in various patient settings, with antioxidants playing a key-role [47,48,49,50,51,52,53]. Surprisingly, our results show that the association of lower plasma vitamin C with cancer mortality risk is independent of fruit and vegetable intake, introducing vitamin C as a specific therapeutic target in this setting of patients.

A possible explanation for the association we found could be the important role that vitamin C plays as epigenetic modulator in health and disease [43,44,45,46], and specifically in cancer cell lines [54]. On the other hand, it is well known that oxidative stress can cause cancer [55,56], due to oxidative damage to deoxyribonucleic acid (DNA) [57]. This oxidative damage is usually counteracted by DNA repair enzymes, but in a pro-oxidant environment, e.g., chronic inflammation and uremic state [58,59], this defense-mechanism is held back [56,60,61]. It has been suggested that antioxidant treatment cannot prevent occurrence of gastrointestinal cancer and that it may even increase overall risk of mortality [55]. However, it has been described that kidney transplant recipients (KTR) have increased oxidative stress [19], which in turn can lead to increased oxidative damage to DNA [57]. Together with decreased immunological surveillance secondary to post-transplant immunosuppression, these phenomena can play a role in increased cancer mortality in KTR and an increased contribution of oxidative stress therein. It can therefore not be excluded that other than subjects of the general population, KTR could benefit from anti-oxidant treatment. High dosages of vitamin C supplementation have been linked to higher risk of development of oxalate kidney stones in male subjects of the general population [62,63]. Vitamin C supplementation may also enhance immunity, which could result in increased risk of rejection. Such effects could limit the utility of vitamin C supplementation in clinical practice and should be taken into account when considering vitamin C supplementation strategies in KTR. Of note, no significant interaction of the association of vitamin C with cancer mortality was found by oxidative stress biomarkers. In light of these results, it could be hypothesized that the inverse association of vitamin C with cancer mortality hereby reported may be explained by its potential role as epigenetic modulator rather than through its antioxidant properties. The latter may be further supported by the finding that plasma vitamin C was inversely associated with cancer mortality independently of fruit and vegetable intake, which suggests that the beneficial effect of vitamin C would not be fully related to the classic theory of dietary intake of natural antioxidants as anticarcinogens [53,57].

Our study has important strengths, including its large sample size of stable KTR, which were closely monitored during a considerable follow-up period by regular check-up in the outpatient clinic, without loss of participants to follow-up. Furthermore, data were extensively collected, allowing to adjust our findings for several potential confounders and predictors of the main results, including current or former smoking status. We acknowledge the study’s limitations as the following. First, vitamin C was measured at baseline. Like the current study, most epidemiological studies use a single baseline measurement to predict outcomes, which adversely affects predictive properties of variables associated with outcomes [64,65,66,67]. If intra-individual variability of predictive biomarkers using repeated measurements is taken into account, this results in strengthening of predictive properties, particularly in case of markers with high intra-individual variation [64,67]. The lower the intra-individual variation from one measurement to the next would be, the more accurate the single measurement represents the usual level of the marker [64,65,66,67]. Noteworthy, evidence available for intra-individual variability of plasma vitamin C suggests that its concentrations relatively stable over time, with a single plasma vitamin C measurement being representative of an individual’s status for long periods of time [65]. Moreover, previous epidemiological studies have used a baseline measurement of plasma vitamin C to predict clinical outcomes over a period of several years [68,69,70]. Second, we measured plasma vitamin C rather than leukocyte vitamin C, which could have provided assessment of tissue vitamin C, and therefore additional information on the role of vitamin C in disease prevention [71]. Third, initiation of vitamin C supplementation during follow-up was not recorded, which could have introduced bias that cannot be accounted for in our analyses. Fourth, incidence and types of non-fatal cancer were not documented, while this information would have been of added value to the reported findings. With the presented data, we had no power to discriminate the association with cancer mortality by types of cancer, which does not necessarily imply that associations are similar for all types of cancer. Nevertheless, our results show, for the first time, a prospective association of plasma vitamin C with long-term risk of cancer mortality in stable kidney transplant recipients, which holds a plea for future studies in which data on incidence and types of non-fatal cancer are collected. To allow for such studies we have started a new large, long-lasting prospective cohort study in kidney transplant recipients in which collection of such data is included [72]. Another limitation is that history of cured skin cancer was not documented, it could therefore not be included in multivariable analyses. Finally, due to its observational design, conclusions on causality cannot be drawn from our results.

In conclusion, we show that cancer is a substantially prevalent individual cause of death after kidney transplantation, and that plasma vitamin C concentrations are inversely and independently associated with cancer mortality risk. Remarkably, our findings link for the first time plasma vitamin C concentrations with cancer mortality risk in KTR, which underscores that vitamin C may be a meaningful as yet overlooked modifiable risk factor of cancer mortality in KTR. Considering the relative increase in cancer mortality rates in kidney transplant recipients along with the decline in deaths due to cardiovascular causes, it is expected that novel risk management strategies are to emerge. Whether a novel vitamin C-targeted strategy may represent an opportunity to decrease the burden of cancer mortality in KTR requires further studies.

## Figures and Tables

**Table 1:** Baseline characteristics of 598 kidney transplant recipients.

Baseline Characteristics	All Patients
Study subjects, *n* (%)	598 (100)
Plasma vitamin C, µmol/L, mean (SD)	44 (20)
**Demographics**	
Age, years, mean (SD)	51 (12)
Sex, male, *n* (%)	328 (55)
Caucasian ethnicity, *n* (%)	577 (97)
**Body composition**	
Body mass index, kg/m^2^, mean (SD)	26.0 (4.3)
Body surface area, m^2^, mean (SD)	1.9 (0.2)
Waist circumference, cm, mean (SD) ^a^	97 (14)
**Kidney allograft function**	
estimated Glomerular Filtration Rate, mL/min/1.73 m^2^, mean (SD)	47 (16)
Proteinuria ≥0.5 g/24 h, *n* (%) ^b^	166 (28)
**Tobacco use**	
Never smoker, *n* (%)	214 (36)
Ex-smoker, *n* (%)	251 (42)
Current smoker, *n* (%)	131 (22)
**Blood pressure**	
Systolic blood pressure, mmHg, mean (SD)	153 (23)
Diastolic blood pressure, mmHg, mean (SD)	90 (10)
**Prior history of cardiovascular disease**	
History of myocardial infarction, *n* (%) ^c^	48 (8)
History of cerebrovascular accident or transient ischemic attack, *n* (%) ^c^	32 (5)
**Diet**	
Fruit intake, servings/day, mean (SD) ^d^	1.5 (1.0)
Vegetable intake, tablespoons/day, mean (SD) ^d^	2.5 (0.8)
**Diabetes and glucose homeostasis**	
Diabetes, *n* (%)	105 (18)
HbA_1C_, %, mean (SD) ^a^	6.5 (1.1)
Insulin, µU/mL, median (IQR)	11.2 (8.0–16.3)
Glucose, mmol/L, median (IQR)	4.5 (4.1–5.0)
**Laboratory measurements**	
Leukocyte concentration, × 10^9^/L, mean (SD) ^b^	8.6 (2.4)
hs-CRP, mg/L, median (IQR)	2.0 (0.8–4.8)
Albumin, g/L, mean (SD) ^a^	41 (3)
**Lipids**	
Total cholesterol, mmol/L, mean (SD)	5.6 (1.1)
HDL cholesterol, mmol/L, mean (SD)	1.1 (0.3)
LDL cholesterol, mmol/L, mean (SD)	3.5 (1.0)
Free fatty acids, µmol/L, mean (SD) ^e^	403 (180)
Triglycerides, mmol/L, median (IQR)	1.9 (1.4–2.6)
**Oxidative stress**	
Uric acid, mmol/L, mean (SD) ^f^	0.45 (0.13)
Malondialdehyde, µmol/L, mean (SD) ^b^	5.6 (1.8)
Gamma-glutamyl transpeptidase, U/L, median (IQR) ^c^	24 (18–39)
Alkaline phosphatase, U/L, median (IQR) ^a^	72 (57–94)
**Kidney transplant and immunosuppressive therapy**	
Dialysis vintage, months, median (IQR)	27 (13–48)
Time since transplantation, years, median (IQR)	6 (3–11)
Donor type (living), *n* (%)	83 (14)
Use of calcineurin inhibitor, *n* (%)	470 (79)
Cyclosporine, *n* (%)	386 (65)
Tacrolimus, *n* (%)	84 (14)
Use of antimetabolites, *n* (%)	441 (74)
Azathioprine, *n* (%)	194 (32)
Mycophenolate acid, *n* (%)	247 (41)
Use of mammalian target of rapamycin inhibitors, *n* (%)	10 (1.7)
Cumulative dose of prednisolone, g, median (IQR) ^b^	21 (11–38)

Data available in: ^a^ 597, ^b^ 596, ^c^ 594, ^d^ 400, ^e^ 471, ^f^ 595. Abbreviations: hs-CRP, high-sensitive C reactive protein; HDL, high-density lipoprotein; IQR, interquartile range; LDL, low-density lipoprotein; HbA_1C_, glycated hemoglobin; SD, standard deviation.

**Table 2:** Association of baseline characteristics with plasma vitamin C in 598 kidney transplant recipients.

Baseline Characteristics	Plasma Vitamin C (Log_2_), µmol/L	
	Linear Regression ^†^	Backwards Linear Regression ^§^
	Std. β	Std. β
Study subjects, *n* (%)	―	―
Plasma vitamin C, µmol/L, mean (SD)	―	―
**Demographics**		
Age, years	−0.56	
Sex, male	−0.19 ***	−0.18 ***
Caucasian ethnicity	−0.21	
**Body composition**		
Body mass index, kg/m^2^	−0.08 *	^~^
Body surface area, m^2^	−0.06	
Waist circumference, cm	−0.15 ***	^~^
**Kidney allograft function**		
estimated Glomerular Filtration Rate, mL/min/1.73 m^2^	0.11 ***	^~^
Proteinuria ≥0.5 g/24 h	−0.11 ***	−0.11 **
**Tobacco use**		
Never smoker	0.03	
Ex-smoker	0.08 *	^~^
Current smoker	−0.11 ***	^~^
**Blood pressure**		
Systolic blood pressure, mmHg	−0.12 ***	^~^
Diastolic blood pressure, mm Hg	−0.1 **	−0.16 ***
**Prior history of cardiovascular disease**		
History of myocardial infarction	−0.01	
History of cerebrovascular accident or transient ischemic attack	−0.04	
**Diet**		
Fruit intake, servings/day	0.22 ***	0.22 ***
Vegetable intake, tablespoons/day	0.09 *	^~^
**Diabetes and glucose homeostasis**		
Diabetes	−0.11 ***	^~^
HbA_1C_, %	−0.13 ***	−0.14 ***
Insulin, µU/mL	−0.09 **	^~^
Glucose, mmol/L	−0.07 *	^~^
**Laboratory measurements**		
Leukocyte concentration, x × 10^9^/L	−0.03	
hs-CRP, mg/L	−0.14 ***	−0.17 ***
Albumin, g/L	0.14 ***	^~^
**Lipids**		
Total cholesterol, mmol/L	0.05	
HDL cholesterol, mmol/L	0.12 ***	^~^
LDL cholesterol, mmol/L	0.07 *	^~^
Free fatty acids, µmol/L	−0.07	
Triglycerides, mmol/L	−0.09 **	^~^
**Oxidative stress**		
Uric acid, mmol/L	−0.14 ***	^~^
Malondialdehyde, µmol/L	0.01	
Gamma-glutamyl transpeptidase, U/L	−0.05	
Alkaline phosphatase, U/L	−0.18 ***	−0.15 ***
**Kidney transplant and immunosuppressive therapy**		
Dialysis vintage, months	−0.09 **	−0.09 **
Time since transplantation, years	0.18 ***	^~^
Donor type (living)	0.02	
Use of calcineurin inhibitor	−0.08 **	^~^
Cyclosporine	−0.03	
Tacrolimus	−0.06	
Use of antimetabolites	0.01	
Azathioprine	0.10 **	^~^
Mycophenolate acid	−0.09 **	^~^
Use of mammalian target of rapamycin inhibitors	−0.09 **	^~^
Cumulative dose of prednisolone, g	0.17 ***	^~^

* *p* Value < 0.1; ** *p* Value < 0.05; *** *p* Value < 0.01. ^†^ Linear regression analysis; adjusted for age and sex. ^§^ Stepwise backwards linear regression analysis; for inclusion and exclusion in this analysis, *p* Values were set at 0.1 and 0.05, respectively. ^~^ Excluded from the final model. Abbreviations: Std. β, standardized beta coefficient; hs-CRP, high-sensitive C reactive protein; HDL, high-density lipoprotein; LDL, low-density lipoprotein; HbA_1C_, glycated hemoglobin.

**Table 3:** Association of plasma vitamin C with cancer mortality in 598 kidney transplant recipients.

Models	Vitamin C (Log_2_), Continuous (µmol/L)		
	HR ^a^	95% CI	*p* Value
Crude	0.63	0.43–0.92	0.016
Model 1	0.61	0.43–0.87	0.006
Model 2	0.52	0.35–0.75	0.001
Model 3	0.50	0.34–0.74	<0.001
Model 4	0.49	0.33–0.72	<0.001
Model 5	0.55	0.38–0.80	0.002
Model 6	0.47	0.32–0.70	<0.001

Cox proportional hazards regression analyses were performed to assess the association of plasma vitamin C with cancer mortality. Model 1: adjustment for age, sex and smoking status. Model 2: Model 1 + adjustment for estimated Glomerular Filtration Rate, dialysis vintage, time since transplantation and proteinuria. Model 3: Model 2 + adjustment for fruit and vegetable intake. Model 4: Model 3 + adjustment for diabetes mellitus, high-sensitivity C-reactive protein and prior history of cardiovascular disease. Model 5: Model 3 + adjustment for immunosuppressive therapy. Model 6: Model 3 + adjustment for transplantation era. Abbreviations: HR, hazard ratio; CI, confidence interval. ^a^ Each model hazard ratio is given per doubling of vitamin C concentration.

**Table 4:** Association of plasma vitamin C with cardiovascular mortality in 598 kidney transplant recipients.

Models	Vitamin C (Log_2_), Continuous (µmol/L)		
	HR	95% CI	*p* Value
Crude	0.97	0.70–1.33	0.83
Model 1	0.97	0.71–1.33	0.86
Model 2	1.04	0.75–1.44	0.83
Model 3	1.16	0.83–1.62	0.40
Model 4	1.31	0.92–1.86	0.13
Model 5	1.21	0.86–1.70	0.27
Model 6	1.15	0.82–1.61	0.41

Cox proportional hazards regression analyses were performed to assess the association of plasma vitamin C with cardiovascular mortality. Model 1: adjustment for age, sex, and smoking status. Model 2: Model 1 + adjustment for estimated Glomerular Filtration Rate, dialysis vintage, time since transplantation and proteinuria. Model 3: Model 2 + adjustment for fruit and vegetable intake. Model 4: Model 3 + adjustment for diabetes mellitus, high-sensitivity C-reactive protein and prior history of cardiovascular disease. Model 5: Model 3 + adjustment for immunosuppressive therapy. Model 6: Model 3 + adjustment for transplantation era. Abbreviations: HR, hazard ratio; CI, confidence interval.

## Supplementary Materials

The following are available online at https://www.mdpi.com/2077-0383/8/12/2064/s1, Figure S1: Strobe flow diagram, Table S1: Death-related type of cancer, Table S2: Association of plasma vitamin C with cancer mortality, all models, Table S3: Interaction analyses for potential confounders on the association of vitamin C with cancer mortality, Table S4: Sensitivity analysis; association of plasma vitamin C with cancer mortality in 598 kidney transplant recipients, censored for graft-failure, Table S5: Sensitivity analysis; association of plasma vitamin C with cardiovascular mortality in 598 kidney transplant recipients, censored for graft-failure, Table S6: Sensitivity analysis; association of plasma vitamin C with cancer mortality in 598 kidney transplant recipients, with HbA1c instead of diabetes mellitus as potential confounder.
