# Supplementary material for: Plasma Vitamin C and Cancer Mortality in Kidney Transplant Recipients
Source: J Clin Med. 2019 Nov 23;8(12):2064. doi: 10.3390/jcm8122064 (PMC6947225; doi:10.3390/jcm8122064)

TABLE OF CONTENTS

Table S1. Death-related type of cancer.

Table S2. Association of plasma vitamin C with cancer mortality, all models.

Table S3. Interaction analyses for potential confounders on the association of vitamin C with cancer mortality.

Table S4. Sensitivity analysis; association of plasma vitamin C with cancer mortality in 598 kidney transplant recipients, censored for graft-failure.

Table S5. Sensitivity analysis; association of plasma vitamin C with cardiovascular mortality in 598 kidney transplant recipients, censored for graft-failure.

Table S6. Sensitivity analysis; association of plasma vitamin C with cancer mortality in 598 kidney transplant recipients, with HbA1c instead of diabetes mellitus as potential confounder.

Figure S1. Strobe flow diagram.

TABLES

Supplemental Table 1. Death-related type of cancer.

Type of cancer	Number of events
Breast cancer	2
Post-transplant lymphoproliferative disorder	2
Small cell lung cancer	2
Unknown primary	1
Bladder carcinoma	1
T-cell acute lymphocytic leukemia	1
Anaplastic oligodendroglioma	1
Parotid cancer	1
Undifferentiated bronchial carcinoma	1
Myelodysplastic syndrome	1
Glioblastoma multiforme	1
Prostate cancer	1
Pancreatic head carcinoma	1
Undifferentiated synovial sarcoma	1
Squamous cell carcinoma, metastatic	1
Ovarian carcinoma, metastatic	1
Renal cell carcinoma	1
Hepatocellular carcinoma	1
Merkel cell carcinoma	1
Colorectal adenocarcinoma	1
Hodgkin lymphoma	1
Esophageal adenocarcinoma	1
Not available*	7

Supplemental Table 2. Primary prospective analysis. Cox proportional hazards regression of cancer mortality, all models.

Variables in the equation	HR	95% CI	*P* Value
**Crude**			
Vitamin C, μmol/L	0.63	0.43-0.92	0.02
**Model 1**			
Vitamin C, μmol/L	0.61	0.43-0.87	0.01
Age, years	1.06	1.03-1.10	0.01
Sex, male	0.36	0.17-0.76	0.01
Smoking status	1.47	0.69-3.15	0.32
**Model 2**			
Vitamin C, μmol/L	0.52	0.35-0.75	0.01
Age, years	1.06	1.03-1.10	0.01
Sex, male	0.31	0.14-0.67	0.01
Smoking status	1.58	0.72-3.48	0.25
estimated Glomerular Filtration Rate, mL/min/1.73 m^2	1.00	0.98-1.02	0.98
Dialysis vintage, months	0.98	0.96-0.99	0.02
Time since transplantation, years	1.02	0.97-1.07	0.51
Proteinuria ≥0.5 g/24 h	0.93	0.40-2.16	0.87
**Model 3**			
Vitamin C, μmol/L	0.50	0.34-0.74	<0.001
Age, years	1.06	1.03-1.10	0.01
Sex, male	0.29	0.13-0.65	0.01
Smoking status	1.50	0.67-3.34	0.32
estimated Glomerular Filtration Rate, mL/min/1.73 m^2	1.00	0.98-1.03	0.88
Dialysis vintage, months	0.98	0.96-1.00	0.02
Time since transplantation, years	1.02	0.97-1.08	0.46
Proteinuria ≥0.5 g/24 h	1.00	0.42-2.36	0.99
Fruit intake, servings/day	1.04	0.78-1.38	0.80
Vegetable intake, tablespoons/day	1.28	0.91-1.79	0.15
**Model 4**			
Vitamin C, μmol/L	0.49	0.33-0.72	<0.001
Age, years	1.06	1.03-1.10	0.01
Sex, male	0.29	0.13-0.66	0.01
Smoking status	1.52	0.67-3.44	0.32
estimated Glomerular Filtration Rate, mL/min/1.73 m^2	1.00	0.97-1.02	0.90
Dialysis vintage, months	0.98	0.96-1.00	0.01
Time since transplantation, years	1.02	0.97-1.08	0.49

Proteinuria ≥0.5 g/24 h	0.98	0.41-2.33	0.96
Fruit intake, servings/day	1.01	0.75-1.35	0.96
Vegetable intake, tablespoons/day	1.32	0.93-1.87	0.12
Diabetes mellitus	1.10	0.48-2.53	0.82
hs-CRP, mg/L	1.00	0.97-1.03	0.85
History of myocardial infarction	0.24	0.03-1.93	0.18
History of cerebrovascular accident or transient ischemic attack	1.52	0.44-5.34	0.51
**Model 5**			
Vitamin C, μmol/L	0.55	0.38-0.80	0.002
Age, years	1.07	1.03-1.11	0.01
Sex, male	0.28	0.12-0.63	0.01
Smoking status	1.50	0.65-3.46	0.34
estimated Glomerular Filtration Rate, mL/min/1.73 m^2	1.00	0.98-1.03	0.97
Dialysis vintage, months	0.97	0.96-0.99	0.01
Time since transplantation, years	0.85	0.70-1.03	0.10
Proteinuria ≥0.5 g/24 h	0.78	0.32-1.91	0.59
Fruit intake, servings/day	0.90	0.66-1.23	0.51
Vegetable intake, tablespoons/day	1.38	0.96-1.98	0.08
Use of cyclosporine	0.31	0.11-0.91	0.03
Use of tacrolimus	0.36	0.08-1.76	0.21
Use of azathioprine	1.90	0.60-5.97	0.28
Use of mycophenolic acid	0.61	0.17-2.17	0.45
Use of mammalian target of rapamycin inhibitors	0.00	0.00-1.61	0.97
Cumulative dose of prednisolone, g	1.03	0.98-1.09	0.28
**Model 6**			
Vitamin C, μmol/L	0.47	0.32-0.70	<0.001
Age, years	1.06	1.03-1.10	0.001
Sex, male	0.27	0.12-0.63	0.002
Smoking status	1.56	0.70-3.51	0.28
estimated Glomerular Filtration Rate, mL/min/1.73 m^2	1.00	0.98-1.03	0.80
Dialysis vintage, months	0.98	0.96-1.00	0.02
Time since transplantation, years	0.95	0.83-1.08	0.40
Proteinuria ≥0.5 g/24 h	0.90	0.37-2.15	0.81
Fruit intake, servings/day	1.04	0.78-1.40	0.80
Vegetable intake, tablespoons/day	1.29	0.92-1.82	0.15
Transplantation era 1968-1989			0.32
Transplantation era 1989-1996	2.50	0.76-8.27	0.13
Transplantation era 1997-2002	4.64	0.49-43.6	0.18

Supplemental Table 3. Interaction analyses for potential confounders on the association of vitamin C with cancer mortality.

Potential confounders	Vitamin C (Log_2), continuous (µmol/L)
	P_{int}
Age, years	0.43
Sex, male	0.64
estimated Glomerular Filtration Rate, mL/min/1.73 m^2	0.23
Time since transplantation, years	0.97
Dialysis vintage, months	0.84
Fruit intake, servings/day	0.56
Vegetable intake, tablespoons/day	0.29
Current or ex-smoker	0.45
hs-CRP, mg/L	0.60
Use of cyclosporine	0.13
Use of tacrolimus	0.16
Use of azathioprine	0.20
Use of mycophenolic acid	0.21
Use of mammalian target of rapamycin inhibitors	1.00
Cumulative dose of prednisolone, g	0.86
Transplantation era	0.99

Supplemental Table 4. Sensitivity analysis; association of plasma vitamin C with cancer mortality in 598 kidney transplant recipients, censored for graft failure.

**Models**	**Vitamin C (Log_2), continuous (μmol/L)**		
	**HR**	**95% CI**	***P* Value**
**Crude**	0.66	0.45–0.97	0.034
**Model 1**	0.63	0.44–0.91	0.014
**Model 2**	0.53	0.36–0.78	0.001
**Model 3**	0.51	0.34–0.76	0.001
**Model 4**	0.50	0.33–0.76	0.001
**Model 5**	0.56	0.38–0.82	0.003
**Model 6**	0.49	0.32-0.73	0.001

Cox proportional hazards regression analyses were performed to assess the association of plasma vitamin C with cancer mortality. Model 1: adjustment for age, sex and smoking status. Model 2: Model 1 + adjustment for estimated Glomerular Filtration Rate, dialysis vintage, time since transplantation and proteinuria. Model 3: Model 2 + adjustment for fruit and vegetable intake. Model 4: Model 3 + adjustment for diabetes mellitus, high-sensitivity C-reactive protein and prior history of cardiovascular disease. Model 5: Model 3 + adjustment for immunosuppressive therapy. Model 6: Model 3 + adjustment for transplantation era. Abbreviations: HR, hazard ratio; CI, confidence interval.

Supplemental Table 5. Sensitivity analysis; association of plasma vitamin C with cardiovascular mortality in 598 kidney transplant recipients, censored for graft failure.

Models	Vitamin C (Log_2), continuous (μmol/L)		
	HR	95% CI	*P* Value
**Crude**	0.96	0.69–1.34	0.80
**Model 1**	0.98	0.71–1.37	0.93
**Model 2**	1.04	0.74–1.48	0.81
**Model 3**	1.19	0.83–1.71	0.34
**Model 4**	1.36	0.93–1.97	0.11
**Model 5**	1.24	0.86–1.77	0.25
**Model 6**	1.19	0.83-1.70	0.34

Cox proportional hazards regression analyses were performed to assess the association of plasma vitamin C with cardiovascular mortality. Model 1: adjustment for age, sex and smoking status. Model 2: Model 1 + adjustment for estimated Glomerular Filtration Rate, dialysis vintage, time since transplantation and proteinuria. Model 3: Model 2 + adjustment for fruit and vegetable intake. Model 4: Model 3 + adjustment for diabetes mellitus, high-sensitivity C-reactive protein and prior history of cardiovascular disease. Model 5: Model 3 + adjustment for immunosuppressive therapy. Model 6: Model 3 + adjustment for transplantation era. Abbreviations: HR, hazard ratio; CI, confidence interval.

Supplemental Table 6. Sensitivity analysis; association of plasma vitamin C with cancer mortality in 598 kidney transplant recipients, with HbA1c instead of diabetes mellitus as potential confounder.

**Models**	**Vitamin C (Log$_2$), continuous (μmol/L)**		
	**HR[a]**	**95% CI**	***P* Value**
**Crude**	0.63	0.43–0.92	0.016
**Model 1**	0.61	0.43–0.87	0.006
**Model 2**	0.52	0.35–0.75	0.001
**Model 3**	0.50	0.34–0.74	<0.001
**Model 4**	0.48	0.32–0.71	<0.001
**Model 5**	0.55	0.38–0.80	0.002
**Model 6**	0.47	0.32-0.70	<0.001

Cox proportional hazards regression analyses were performed to assess the association of plasma vitamin C with cancer mortality. Model 1: adjustment for age, sex and smoking status. Model 2: Model 1 + adjustment for estimated Glomerular Filtration Rate, dialysis vintage, time since transplantation and proteinuria. Model 3: Model 2 + adjustment for fruit and vegetable intake. Model 4: Model 3 + adjustment for HbA1c, high-sensitivity C-reactive protein and prior history of cardiovascular disease. Model 5: Model 3 + adjustment for immunosuppressive therapy. Model 6: Model 3 + adjustment for transplantation era. Abbreviations: HR, hazard ratio; CI, confidence interval.

[a] Each model hazard ratio is given per doubling of vitamin C concentration.

Supplemental Figure 1. STROBE flow diagram.

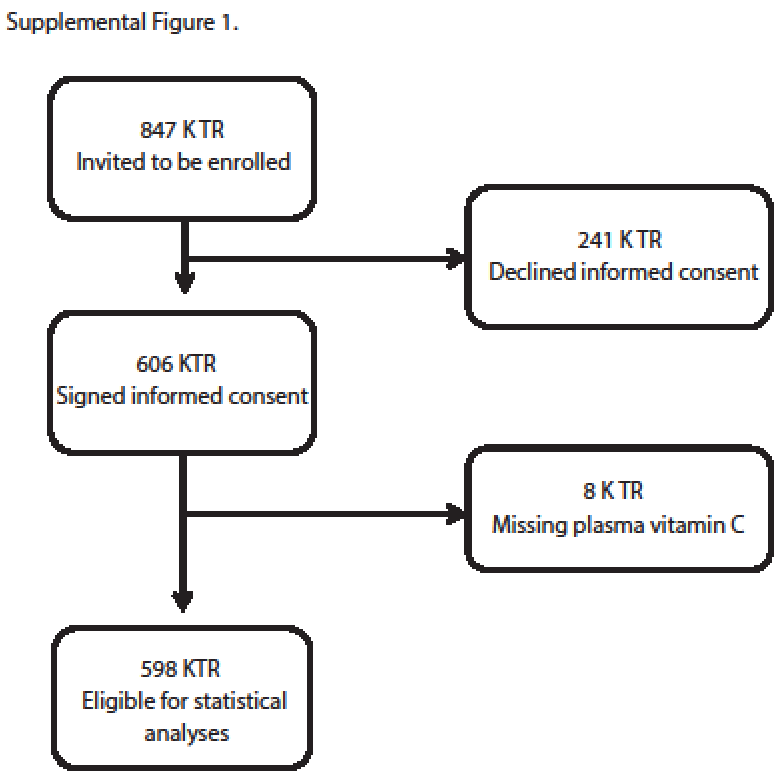

Supplement: Supplementary file 1 [file jcm-08-02064-s001.pdf]
